# Sir2D, a Sirtuin family protein, regulates adenylate cyclase A expression through interaction with the MybB transcription factor early in *Dictyostelium* development upon starvation

**DOI:** 10.1016/j.heliyon.2019.e01301

**Published:** 2019-03-07

**Authors:** Hideo Taniura, Shuhei Soeda, Tomoko Ohta, Maya Oki, Risako Tsuboi

**Affiliations:** Laboratory of Neurochemistry, College of Pharmacy, Ritsumeikan University, Shiga, Japan

**Keywords:** Cell biology, Developmental biology

## Abstract

Sirtuin interacts with many regulatory proteins involved in energy homeostasis, DNA repair, cell survival, and lifespan extension. We investigated the functional roles of Sir2D during early *Dictyostelium* development upon starvation. We found that ectopic expression of Sir2D accelerated development among three Sirtuins containing highly homologous catalytic domain sequences to mouse Sirt1. Sir2D expression upregulated adenylate cyclase A (aca) mRNA expression 2, 4 and 6 h after starvation. We have previously reported that nicotinamide, a Sirt1 inhibitor, treatment delayed the development and decreased the expression of aca at 4 h after starvation. Sir2D expressing cells showed resistance against the nicotinamide effect. RNAi-mediated Sir2D knockdown cells were generated, and their development was also delayed. Aca expression was decreased 4 h after starvation. Sir2D expression restored the developmental impairment of Sir2D knockdown cells. The induction of aca upon starvation starts with transcriptional activation of MybB. The ectopic expression of MybB accelerated the development and increased the expression of aca 2 and 4 h after starvation but did not restore the phenotype of Sir2D knockdown cells. Sir2D expression had no effects on MybB-null mutant cells during early development. Thus, MybB is necessary for the upregulation of aca by Sir2D, and Sir2D is necessary for the full induction of aca after 4 h by MybB. MybB was coimmunoprecipitated with Sir2D, suggesting an interaction between MybB and Sir2D. These results suggest that Sir2D regulates aca expression through interaction with the MybB transcription factor early in *Dictyostelium* development upon starvation.

## 1 Introduction

Sirtuins have been defined as a family of nicotinamide adenine dinucleotide (NAD)-dependent enzymes that deacetylate lysine residue on various proteins. In mammals, seven homologs Sirt1-Sirt7 have been identified. Among them, Sirt1 is the best studied mammalian NAD-dependent histone deacetylase that downregulates the acetylation levels of many regulatory proteins including p53, FOXO, PPARγ, and PGC-1α, which are involved in energy homeostasis, DNA repair, cell survival, and lifespan extension [1, 2].

The cellular slime mold, *Dictyostelium discoideum,* is an excellent model organism for studying the molecular basis of cell differentiation and signal transduction. Although *Dictyostelium* cells grow vegetative as individual amebae, upon starvation, they gather with neighboring cells to form aggregates consisting of up to 10^5^ cells. Aggregating cells relay extracellular cAMP signals and move to the signaling center by chemotaxis to cAMP. cAMP levels are controlled by the developmentally regulated expression of adenylate cyclase [3]. Adenylate cyclase A (aca) is expressed from the first hours of development [4] and is responsible for cAMP synthesis during the aggregation process. cAMP is used both as a secreted intercellular signal and as an intracellular second messenger in signal transduction. After aggregation, cells behave as more complex multicellular organisms and finally culminate into a fruiting body consisting of spores on top of a supporting stalk [5].

Sirtuins are conserved throughout evolution from archaebacterial to eukaryotes [2]. *Dictyostelium discoideum* encodes at least 5 Sirtuin proteins, Sir2A-Sir2E, that show sequence similarity to human Sirtuins [6, 7]. We have previously reported that nicotinamide (NAM), a Sirt1 inhibitor, treatment delayed development and decreased the expression of aca at 4 h after starvation. Resveratrol (RSV), a sirtuin activator, treatment accelerated the development and increased the expression of aca 4 and 6 h after starvation [8]. The findings suggest that Sirtuins are involved in early *Dictyostelium* development upon starvation. We found that Sir2D regulates aca expression through interaction with a putative transcription factor, MybB in early *Dictyostelium* development upon starvation. Although Lohia et al. already reported that Sir2D played a role in cell differentiation, modulated the expression of both prespore and prestalk genes and participated in the process of autophagy [7], we reported another aspect of Sir2D function here.

## 2 Materials and methods

### 2.1 Growth and development of *Dictyostelium*

*Dictyostelium discoideum* Ax-2 cells were provided by the National BioResources Project (NBPR) of Japan. Ax-2 cells were grown axenically in HL5 medium. For development under submerged culture, the cells were washed twice in Na-K phosphate buffer (10 mmol/L Na-K phosphate buffer, 20 mmol/L MgSO_4_, and 2 mmol/L CaCl_2_, pH 6.1), and 2 × 10^6^ cells/mL cells in Na-K buffer were developed over 24 h [9]. For NAM treatment, 10 mmol/L NAM dissolved in water was added to HL5 axenic growth medium a day before development, and the cells were developed in the presence of NAM at the same concentration.

### 2.2 Cloning and expression of Sir2A, Sir2C, Sir2D, and MybB

The entire coding sequences of Sir2A (DDB_G0283917), Sir2C (DDB_G0284795), Sir2D (DDB_G0289967), and MybB (DDB_G275445) were amplified from cDNA and cloned into pBluescript SK+ using BamHI and XhoI sites. The following primers were used for polymerase chain reaction (PCR) amplification: Sir2A, 5′-tgcggatccatgtacgcagtgaatccaattg-3′ and 5′-gcgctcgagttaatttttaactatttgatt-3′, Sir2C, 5′-tgcggatccatgtcaaaacaaacacaa-3′ and 5′-gcgctcgagttaattatggtttttagaatt, Sir2D, 5′-tgcggatccatgaataagagaagatctttaga-3′ and 5′-gcgctcgagtcaccatttaactttatttaat-3′, and MybB, 5′-tgcggatccatgactgctatattcccaaat-3′ and 5′-gcgctcgagatagtaataattttacagcat-3’. To express Flag-tagged or GFP-tagged Sir2A, Sir2C, Sir2D or MybB, these cDNAs were cloned into pTX-Flag or pTX-GFP expression vectors [10]. Flag or GFP was fused at the N termini of these proteins. The construct was introduced into Ax-2 cells by electroporation and stable transformants were selected with G418. For fluorescence immunocytochemistry, the cells were fixed in 4% formaldehyde for 20 min, permeabilized with methanol, and incubated with anti-Flag M2 monoclonal antibody (Sigma-Aldrich) or anti-GFP rabbit monoclonal antibody (Invitrogen). For 4′6′-diamidino-2-phenylindole dihydrochloride (DAPI, Life Technologies) staining, the fixed cells were treated with 300 nmol/L DAPI to visualize nuclei. The images were taken using a fluorescence microscope (EVOS, ThermoFisher Scientific).

### 2.3 Quantitative reverse transcription-PCR (qRT-PCR)

Total RNA was extracted from vegetative, and 2-, 4- and 6 h-starved Ax-2 cells under submerged culture using TRI reagent (Sigma-Aldrich), and cDNA was synthesized from total RNA. cDNA was used as the templates for PCR. Reverse transcription-PCR (RT-PCR) products were quantified with LightCycler FastStart DNA Master PLUS SYBR Green I (Roche Diagnostics) in a LightCycler instrument (Roche Diagnostics). Melting curves were analyzed to confirm a single species of each PCR product. Actin 15 cDNA was used as an internal standard to quantify the relative expression of each cDNA. Statistical significance was tested using Student's t-test. Primers used for real-time PCR were as follows: actin 15; 5′-taaatccaaaagccaacagag-3′ and 5′-ttggaaagttgagagtgaagc-3′, aca; 5′-ttgcaggtttccaagaata-3′ and 5′-tttgctctactgataccgat-3′, carA; 5′-taaatatgtttccaccagca-3′ and 5′-taccacgacttgaactatatg-3′, rasC; 5′-attccgtgaacaaattttaag-3′ and 5′-gatttggctgatgtttctaaa-3’.

### 2.4 Immunoprecipitation

Combinations of expression vectors for Flag-Sir2D alone, GFP-MybB alone, or both Flag-Sir2D and GFP-MybB were introduced into Ax-2 cells, and the transformants were selected with G418. Cell lysates were prepared in a buffer containing 10 mmol/L PIPES (pH 7.0), 100 mmol/L NaCl, 300 mmol/L sucrose, 3 mmol/L MgCl_2_, 1 mmol/L EGTA, 0.5% Triton X-100 and Complete protease inhibitors (Roche Diagnostics). Aliquots (400 μg of protein) of extracts were incubated for 2 h at 4 °C with anti-GFP rabbit monoclonal antibody (Invitrogen). The antibody-protein complexes were pelleted with protein A-Sepharose (GE Healthcare), separated by 7.5% sodium dodecyl sulfate-polyacrylamide gel electrophoresis (SDS-PAGE), and analyzed by immunoblotting with anti-Flag M2 monoclonal antibody (Sigma-Aldrich).

### 2.5 RNA interference (RNAi) mediated knockdown mutant of Sir2D (Sir2D KD cells)

To knockdown the expression of Sir2D, we constructed an expression vector to express a stem-loop RNA directed against Sir2D mRNA. One fragment containing nucleotides +93 to +1589 of the coding region of Sir2D (+1 at the beginning of the ATG start codon) was cloned in sense orientation into pTX BamHI/Xho sites. Another fragment containing nucleotides +93 to +1048 of the coding region was fused in antisense orientation into the above pTX XhoI/XbaI sites. The Sir2D-RNAi vector was introduced into Ax-2 cells and stable transformants were selected with G418. qRT-PCR was performed to quantify the levels of Sir2D mRNA expression. The primers used were as follows: 5′-ttcattacttacgtgcaaa-3′ and 5′-tcttgaacaaattgatcgc-3’. Actin 15 cDNA was used as an internal standard. Statistical significance was tested using Student's t-test. Expression vectors for GFP-Sir2D or GFP-MybB along with a blasticidin resistant gene (BSR) cassette was introduced into Sir2D KD cells to test their restoring effect against Sir2D knockdown.

### 2.6 Isolation of MybB-null mutant

The MybB-null mutant was generated by gene targeting according to the method described previously [4]. The targeting construct contains a genomic fragment that encompasses the MybB coding region from the position +32 to +2949 (+1 at the beginning of the ATG start codon) in which a BSR cassette was inserted at the SacI site (the position +1501). The MybB gene was amplified by PCR using primers containing PstI and EcoRI sites. The following primers were used for PCR amplification: 5′-tgcctgcagcattttattattgggctagca-3′ and 5′-gcggaattcaatattgataagatgggaaat-3’. The construct was linearized with PstI and EcoRI and introduced into Ax-2 cells. Transformants were selected for blasticidin resistance. Homologous recombination was confirmed by PCR using genomic DNA. Two sets of primers (Wt-F/BSR-R and BSR-F/Wt-R) recognize part of the region that is not present in the targeting construct (Wt-F and Wt-R) and in BSR cassette (BSR-F and BSR-R) (Fig. 8). The primers used were as follows: Wt-F: 5′-tgactgctatattcccaaatt-3′, BSR-R: 5′-tagatgtaaaacagccaaaga-3′, BSR-F: 5′-ttttaaagatttgatgggatt-3′, and Wt-R: 5′-aattggtccgaaataaaac-3’. qRT-PCR was also performed to quantify the levels of MybB mRNA expression. The primers used were as follows: 5′-aattaataattcaatgccaca-3′ and 5′-tacagcattaaaagtggat-3’.

## 3 Results

### 3.1 Cellular localization of Sir2A, Sir2C and Sir2D

**Fig. 1:**
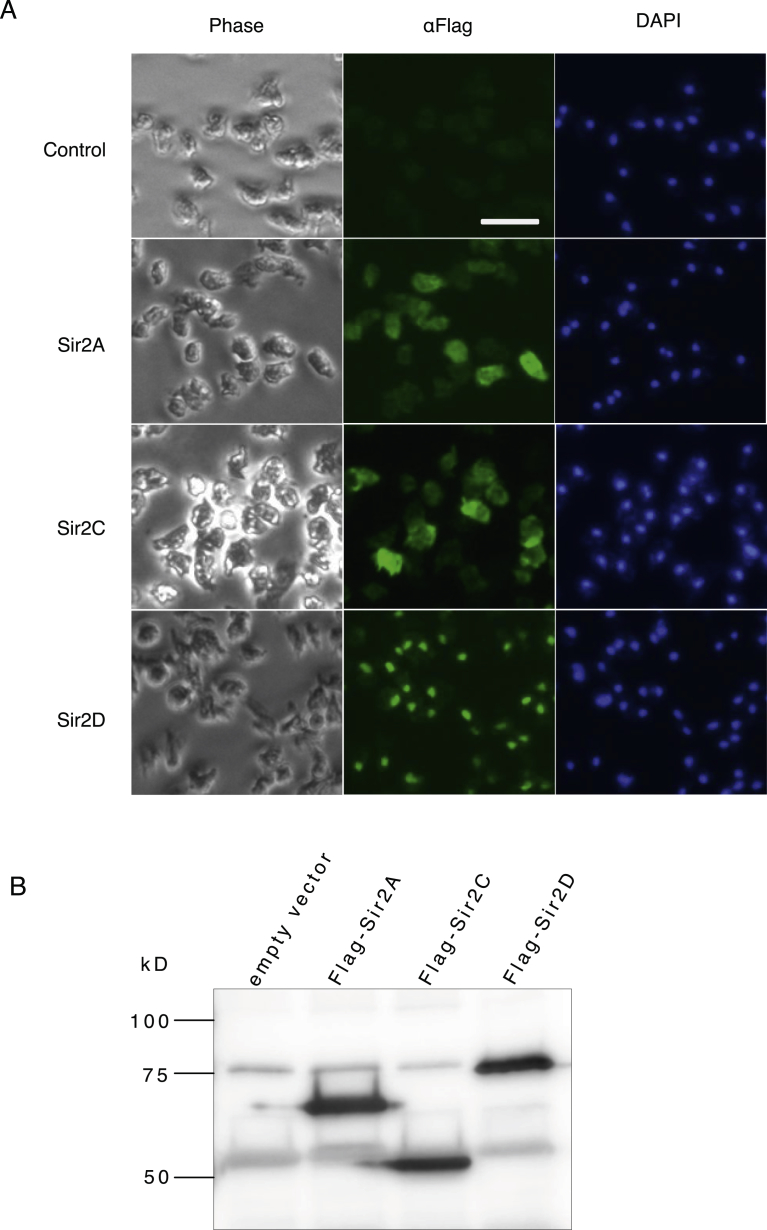
Localization of Sir2A, Sir2C and Sir2D. (A) Localization of Flag-tagged Sir2A, Sir2C and Sir2D in axenically grown Ax-2 cells. Expression of Flag-tagged Sir2A, Sir2C or Sir2D was detected using anti-Flag M2 antibody directed against the Flag epitope under a fluorescence microscope (middle panel, α-Flag). The same cells were also stained with DAPI to show the nuclei (right panel, DAPI). The left panel shows the phase contrast image of the same field of cells (left panel, Phase). Scale bar: 50 μm. (B) Immunoblot of Flag-tagged Sir2A, Sir2C and Sir2D. Cell lysates were prepared from Ax-2 cells expressing Flag-tagged Sir2A, Sir2C or Sir2D and immunoprecipitated with anti-Flag antibody. Flag-tagged fusion proteins were detected by anti-Flag antibody.

The Sirtuin family of proteins contains the highly conserved catalytic core domain composed of a large oxidized NAD-binding Rossmann fold subunit [2]. When the mouse Sirt1 catalytic domain sequence (amino acids 236–490) was compared with *Dictyostelium* Sirtuin sequences, Sir2A, Sir2C and Sir2D sequences showed more than 50% identity with mouse Sirt1 sequence [8], and approximately 10% identity between the mouse Sirt1 and Sir2B or Sir2E was shown. We expressed Sir2A, Sir2C and Sir2D proteins carrying Flag tag at their termini and examined subcellular localization. Flag-tagged Sir2D immunoreactivity was highly enriched in nuclei, whereas Sir2A and Sir2C immunoreactivities were distributed across whole cells (Fig. 1A). Original images are shown in Supplementary Fig. S1A. All the fusion proteins expressed the predicted size products (Fig. 1B). The entire images are shown in Supplementary Fig. S1B.

### 3.2 Sir2D expression accelerates development

**Fig. 2:**
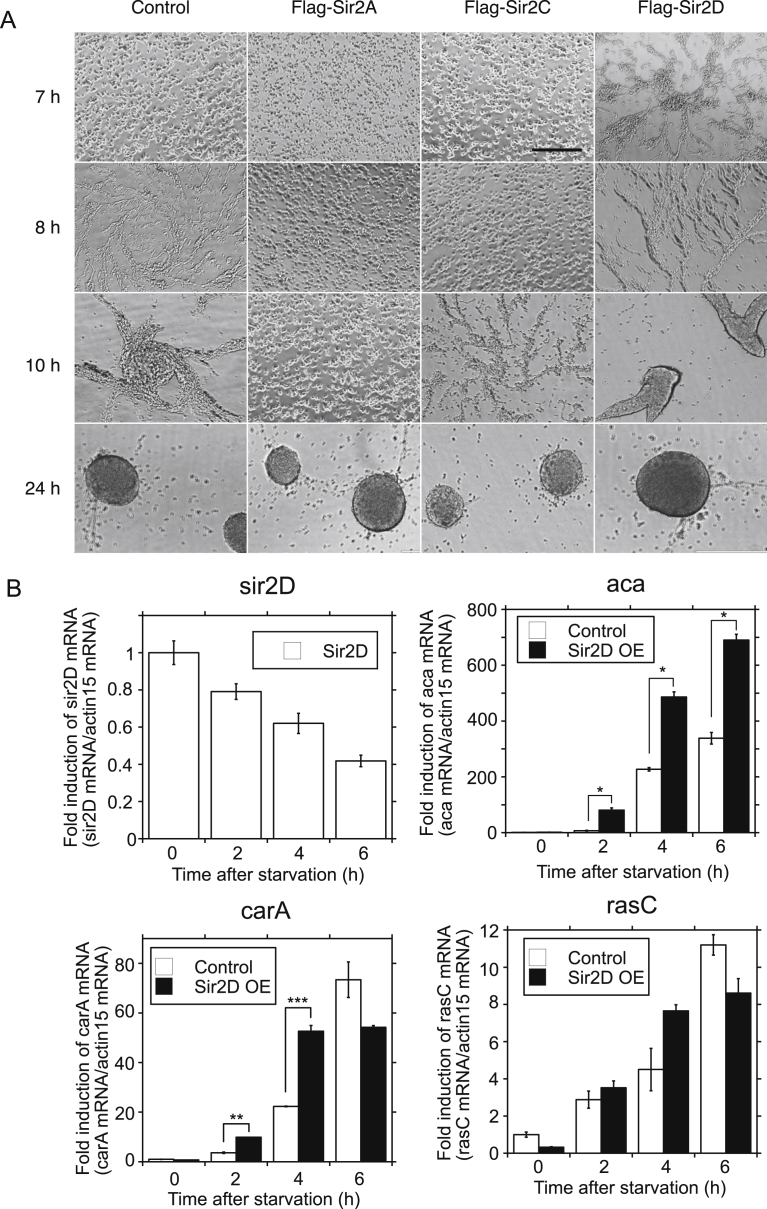
Sir2A, Sir2C and Sir2D OE cell development upon starvation. (A) Control (empty vector), Sir2A, Sir2C and Sir2D OE cells were developed under submerged culture. Development was imaged at 6, 7, 9 and 24 h. Scale bar: 200 μm. (B) Alteration of mRNA expression of aca in Sir2D OE cells during early development upon starvation. Control (empty vector) and Sir2D OE cells were developed under submerged culture. Total RNA was extracted from the cells 0, 2, 4 and 6 h after starvation. qRT-PCR was performed using specific primers for Sir2D, aca, carA, rasC or actin 15. The quantified results were normalized against actin 15 mRNA levels. Each value represents the mean ± standard error (SE) (n = 3). Asterisk indicates a significantly different value (*P < 0.001, **P < 0.005 and ***P < 0.01) from the control's value.

Although *Dictyostelium* cells grow vegetatively as an individual amoeba, upon starvation, they gather with neighboring cells and form aggregates. During this process, cells can be seen streaming toward a central domain aggregation center. To examine the development of Sir2A-, Sir2C- and Sir2D-expressing (OE) cells, we observed the starved cells grown under submerged culture. As shown in Fig. 2A, cell streaming appeared at 7 h, and tight aggregates were formed at 24 h in control cells. Sir2D expression accelerated this development. Cell streaming at 6 h and weak aggregates at 9 h were already observed in Sir2D OE cells (Fig. 2A, Flag-Sir2D). The development process was relatively delayed in Sir2A or Sir2C OE cells (Fig. 2A, Flag-Sir2A and Flag-Sir2C). Original images are shown in Supplementary Fig. S2. Sir2D was expressed in vegetative cells and cells during early development (Fig. 2B, Sir2D). The expression peaked at 0 h and gradually decreased after starvation. The development in *Dictyostelium* upon starvation started inducing the essential molecule, aca, to produce cAMP and a seven transmembrane G protein coupled receptor specific for cAMP (carA) [4, 11]. Transcription of carA from the early, aggregation promoter is activated by cAMP pulsing [12]. One of the responses to extracellular cAMP is the rapid accumulation of active Ras protein on the cell membrane [11]. We examined aca, carA and rasC mRNA expression levels in Sir2D OE cells during the early development by qRT-PCR. As shown in Fig. 2B, aca was induced upon starvation after 4 h in control cells, and Sir2D expression significantly increased the expression of aca 2, 4, and 6 h after starvation by 11.7-, 2.14-, and 2.04-fold compared control cells, respectively. carA expression was also increased significantly at 2 and 4 h. rasC expression was insignificantly increased at 4 h. Thus, the expressions of adenylate cyclase, aca and the signaling molecules carA and rasC were increased by Sir2D overexpression.

### 3.3 Sir2D OE cells withstand delayed development by NAM treatment

**Fig. 3:**
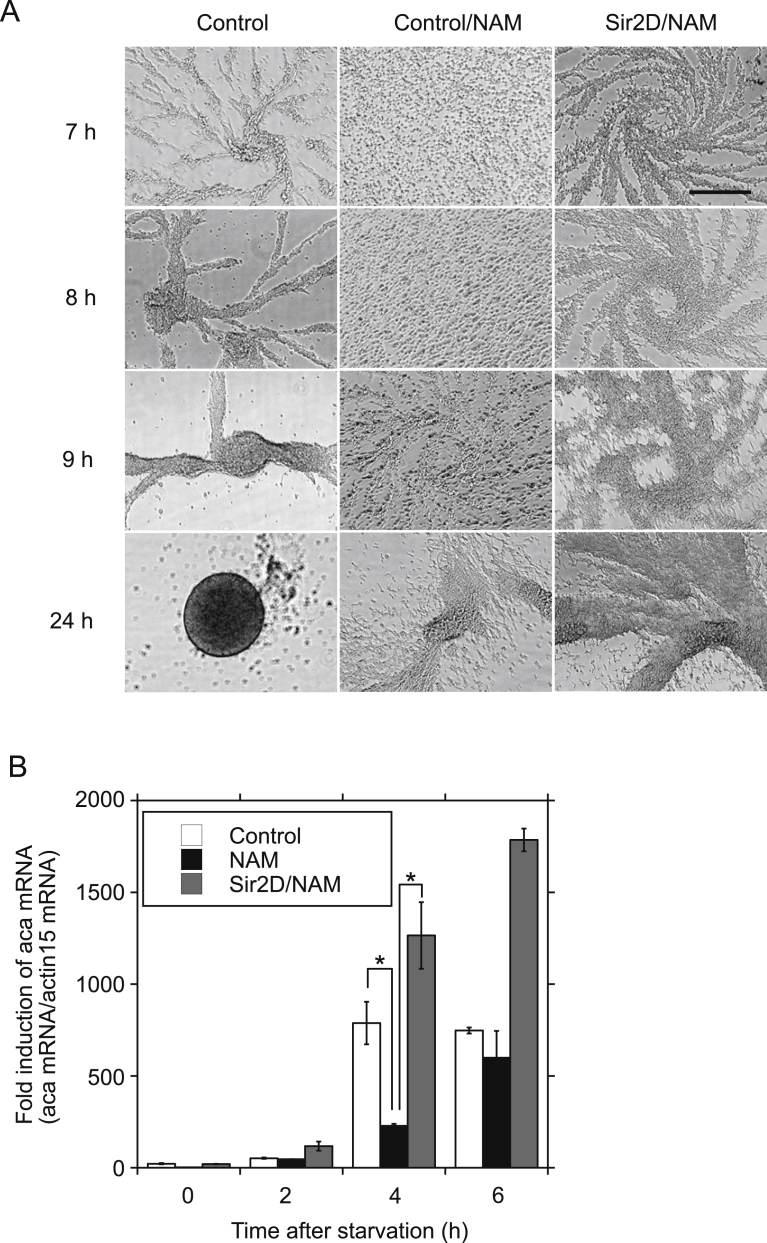
Effect of NAM on cellular development upon starvation. (A) Effect of NAM on cellular development of Sir2D OE cells. Control cells (empty vector) in the presence or absence of NAM (10 mM) were developed under submerged culture (Control and Control/NAM). Sir2D OE cells in the presence of NAM (10 mM) were also developed (Sir2D/NAM). Development was imaged 7, 8, 9 and 24 h after starvation. Scale bar: 200 μm. (B) Effect of NAM on mRNA expression levels of aca. Control cells (empty vector) in the presence or absence of NAM (10 mM) were developed under submerged culture (Control and NAM). Sir2D OE cells in the presence of NAM (10 mM) were also developed (Sir2D/NAM). Total RNA was extracted from the cells 0, 2, 4 and 6 h after starvation. qRT-PCR was performed using specific primers for aca or actin 15. The quantified results were normalized against actin 15 mRNA levels. Each value represents the mean ± standard error (SE) (n = 3). Asterisk indicates a significantly differerent value (**P < 0.01) from the control's value.

As a Sirt1 inhibitor, NAM is commonly used for studying Sirt1 function [13, 14, 15, 16]. We have previously reported that NAM treatment delayed development [8]. The initiation of streaming was delayed at 9 h, while, highly condensed streaming was seen in control cells at the same time (Fig. 3A. Control and Control/NAM). Development was still under cell streaming even after 24 h. When Sir2D OE cells were treated with NAM, the timing of cell streaming was restored comparable to the development of control cells against NAM treatment (Fig. 3A. Control and Sir2D/NAM). However, aggregate formation by Sir2D OE cells was severely impaired at 24 h by NAM treatment. Original images are shown in Supplementary Fig. S3. NAM treatment significantly reduced aca expression level at 4 h to 29% of control cells (Fig. 3B). The reduction in aca expression by NAM treatment at 4 h was restored in Sir2D OE cells. These results suggest that one of the targets of Sirt1 inhibitor, NAM, in *Dictysotelium discoideum* is Sir2D.

### 3.4 RNAi-mediated knockdown of Sir2D expression

**Fig. 4:**
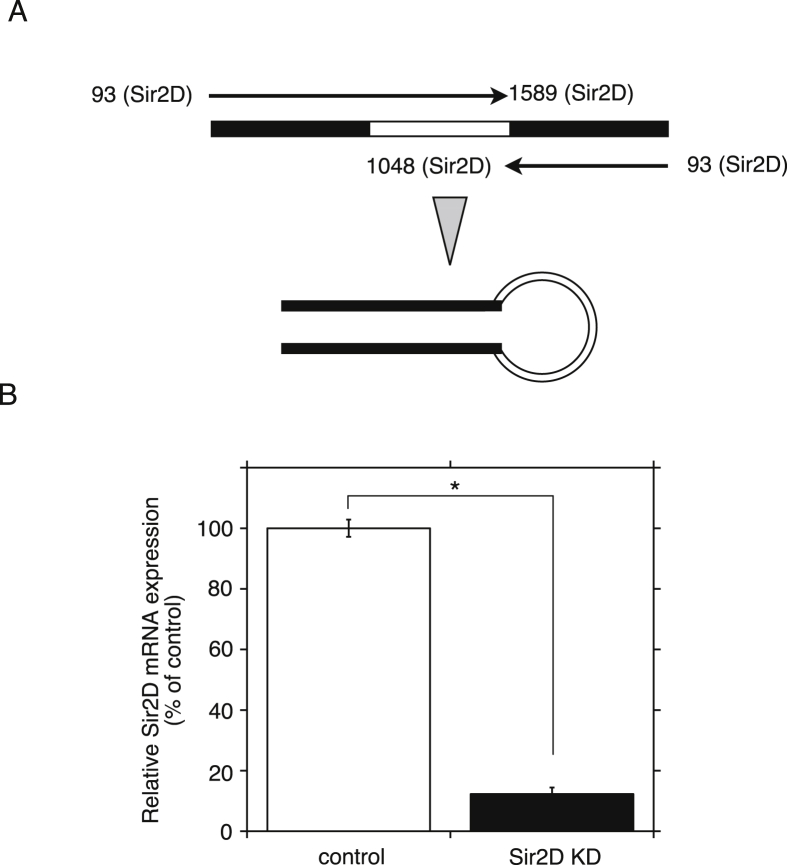
RNAi-mediated knockdown of Sir2D expression. (A) Construction of stem-loop RNA expression directed against Sir2D. One fragment containing nucleotides +93 to +1589 in the coding region of Sir2D (+1 at the beginning of the ATG start codon) and another fragment containing nucleotides +93 to +1048 in the coding region were fused in reverse orientation into the pTX expression vector under the control of the actin 15 promoter. (B) qRT-PCR analysis of Sir2D knockdown cells (Sir2D KD). Total RNA extracted from vegetative control cells (empty vector) and Sir2D KD cells was used for oligo(dT)-primed qRT-PCR. The quantified results were normalized against actin 15 mRNA levels. Each value represents the mean ± standard error (SE) (n = 3). Asterisk indicates a significantly different value (*P < 0.0001) from the control's value.

**Fig. 5:**
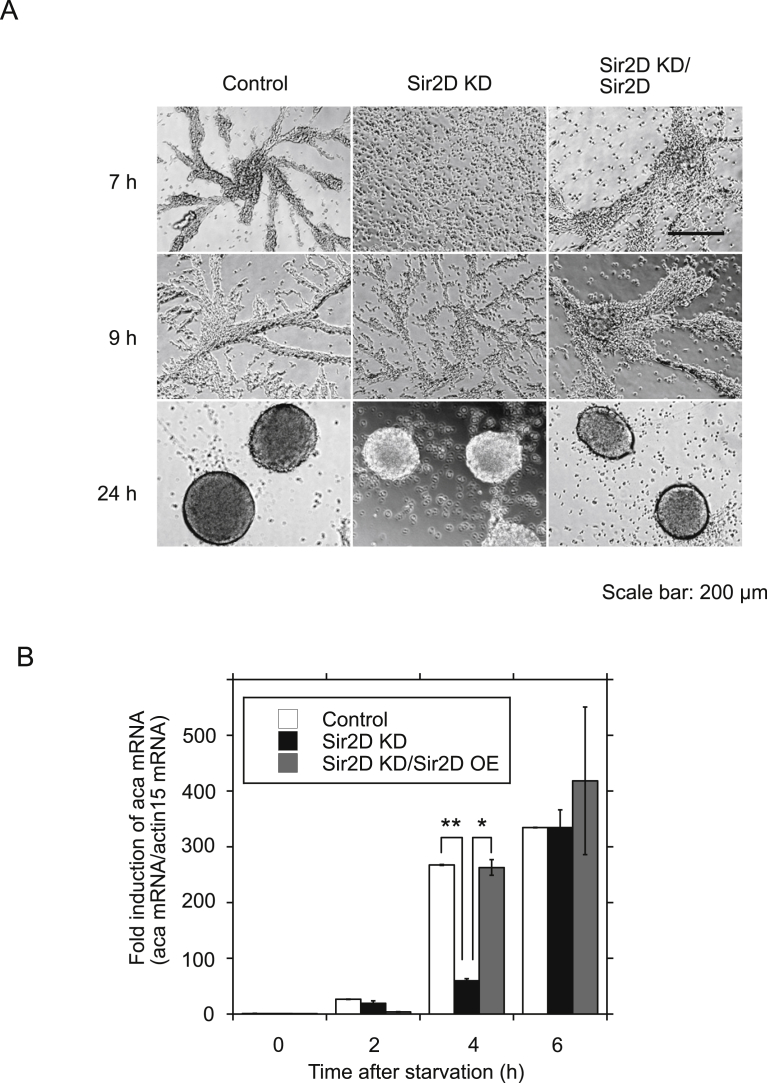
Sir2D KD cells development upon starvation. (A) Cellular development of Sir2D KD cells. Control cells (empty vector) and Sir2D KD cells were developed under submerged culture (Control and Sir2D KD). Sir2D KD cells coexpressed with GFP-Sir2D were also developed (Sir2D KD/Sir2D). Development was imaged 7, 9 and 24 h after starvation. Scale bar: 200 μm. (B) Alteration of mRNA expression of aca in Sir2D KD cells during early development upon starvation. Control cells (empty vector) and Sir2D KD cells were developed under submerged culture (Control and Sir2D KD). Sir2D KD cells coexpressed with GFP-Sir2D were also developed (Sir2D KD/Sir2D OE). Total RNA was extracted from the cells 0, 2, 4 and 6 h after starvation. qRT-PCR was performed using specific primers for aca or actin 15. The quantified results were normalized against actin 15 mRNA levels. Each value represents the mean ± standard error (SE) (n = 3). Asterisk indicates a significantly different value (P*<0.001, **P < 0.0001) from the control's value.

Next, we isolated Sir2D knockdown transformants (Sir2D KD cells) by RNAi. RNAi-mediated gene silencing has been established in *Dictyostelium* [17]. To knockdown the expression of Sir2D, we constructed a plasmid to express a stem-loop RNA directed against Sir2D mRNA under the control of the actin 15 promoter (Fig. 4A). The expression level of Sir2D mRNA in the RNAi expression vector-transfected cells was reduced to 12% of empty vector-transfected control cells (Fig. 4B). Sir2D KD cells showed delayed development upon starvation compared to control cells. The initiation of streaming was delayed at 9 h (Fig. 5A). Original images are shown in Supplementary Fig. S4. Aca expression level was also reduced at 4 h to 22% of control cells (Fig. 5B). This delayed development in Sir2D KD cells was restored in Sir2D KD cells coexpressed with Sir2D (Fig. 5A and B). The developmental impairment of Sir2D KD cells was similar to that of the control cells treated with NAM, except for the impairment of aggregate formation at 24 h. NAM may target another Sirtuin during *Dictyostelium* development.

### 3.5 Interaction of Sir2D with a transcription factor MybB

**Fig. 6:**
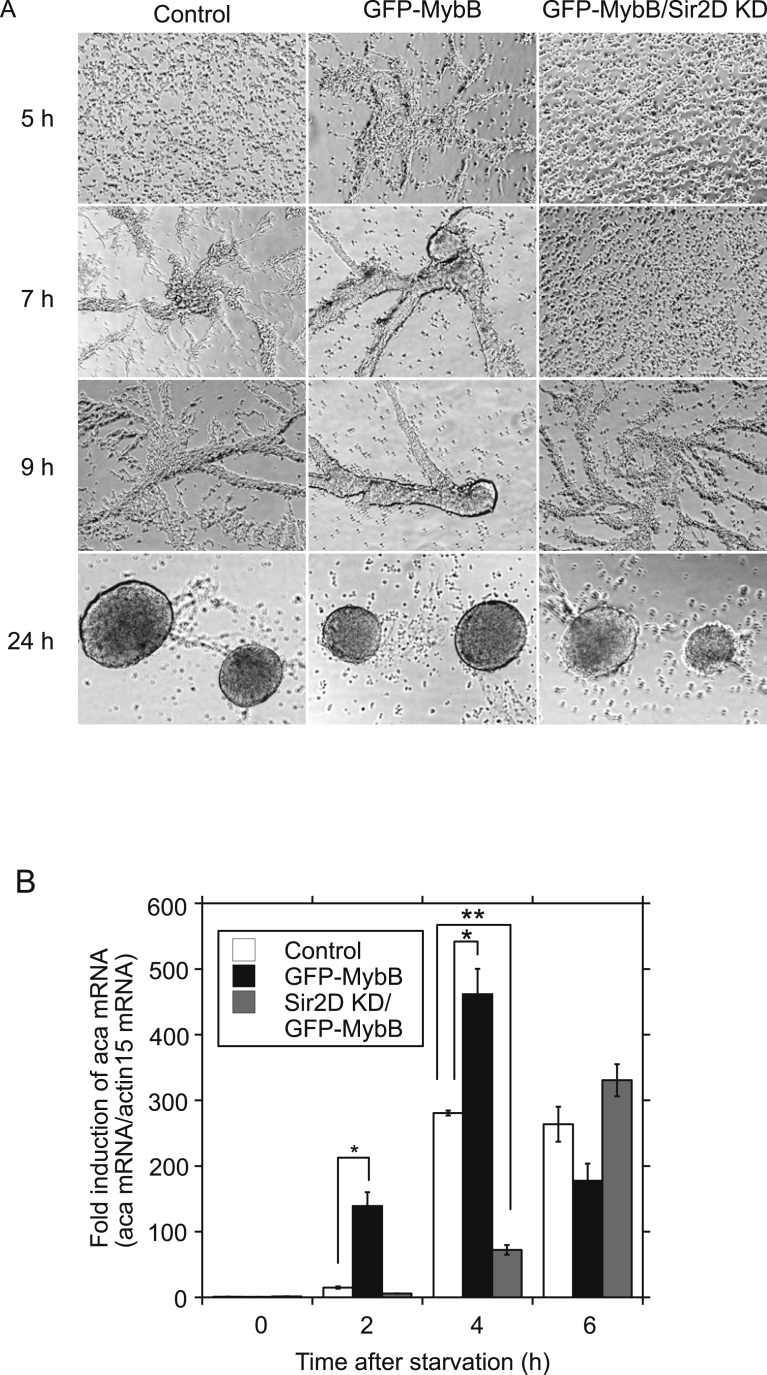
MybB OE cells development upon starvation. (A) Cellular development of MybB OE cells. Control cells (empty vector) and MybB OE cells were developed under submerged culture (Control and GFP-MybB). Sir2D KD cells coexpressed with GFP-MybB were also developed (GFP-MybB/Sir2D KD). Development was imaged 5, 7, 9 and 24 h after starvation. Scale bar: 200 μm. (B) Alteration of mRNA expression of aca in MybB OE cells during early development upon starvation. Control cells (empty vector) and MybB OE cells were developed under submerged culture (Control and GFP-MybB). Sir2D KD cells coexpressed with GFP-MybB were also developed (GFP-MybB/Sir2D KD). Total RNA was extracted from the cells 0, 2, 4 and 6 h after starvation. qRT-PCR was performed using specific primers for aca or actin 15. The quantified results were normalized against actin 15 mRNA levels. Each value represents the mean ± standard error (SE) (n = 3). Asterisk indicates a significantly different value (*P < 0.05, **P < 0.0001) from the control's value.

**Fig. 7:**
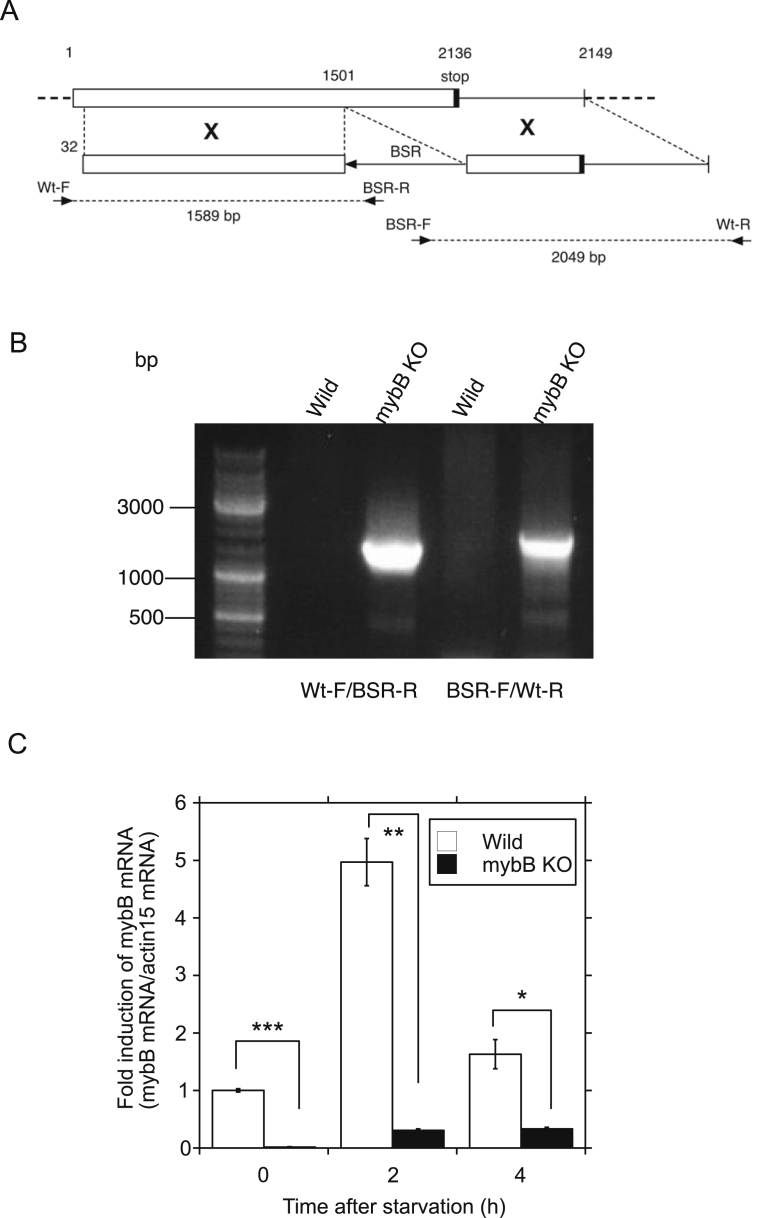
Preparation of MybB knockout cells. (A) Targeting construction. The targeting construct contains a genomic fragment that encompasses the MybB coding region from the position +32 to +2949 (+1 at the beginning of the ATG start codon) in which a BSR cassette was inserted at the SacI site (the position +1501). The arrows denote two primer sets (Wt-F/BSR-R and BSR-F/Wt-R) for PCR analysis and estimated product size was also shown. (B) PCR analysis. Genomic DNA was isolated from wild-type and MybB mutant cells (Wild and MybB KO). PCR analysis was performed using two primer sets of Wt-F/BSR-R and BSR-F/Wt-R. Two primers of Wt-F and Wt-R recognize part of the region that is not present in the targeting construct and BSR-F and BSR-R are in the BSR cassette. (C) qRT-PCR analysis of MybB KO cells (MybB KO). Total RNA extracted from wild-type and MybB KO cells 0, 2 and 4 h after starvation was used for oligo(dT)-primed qRT-PCR. The quantified results were normalized against actin 15 mRNA levels. Each value represents the mean ± standard error (SE) (n = 3). Asterisk indicates a significantly different value (*P < 0.05, **P < 0.001 and ***P < 0.0001) from the control's value.

**Fig. 8:**
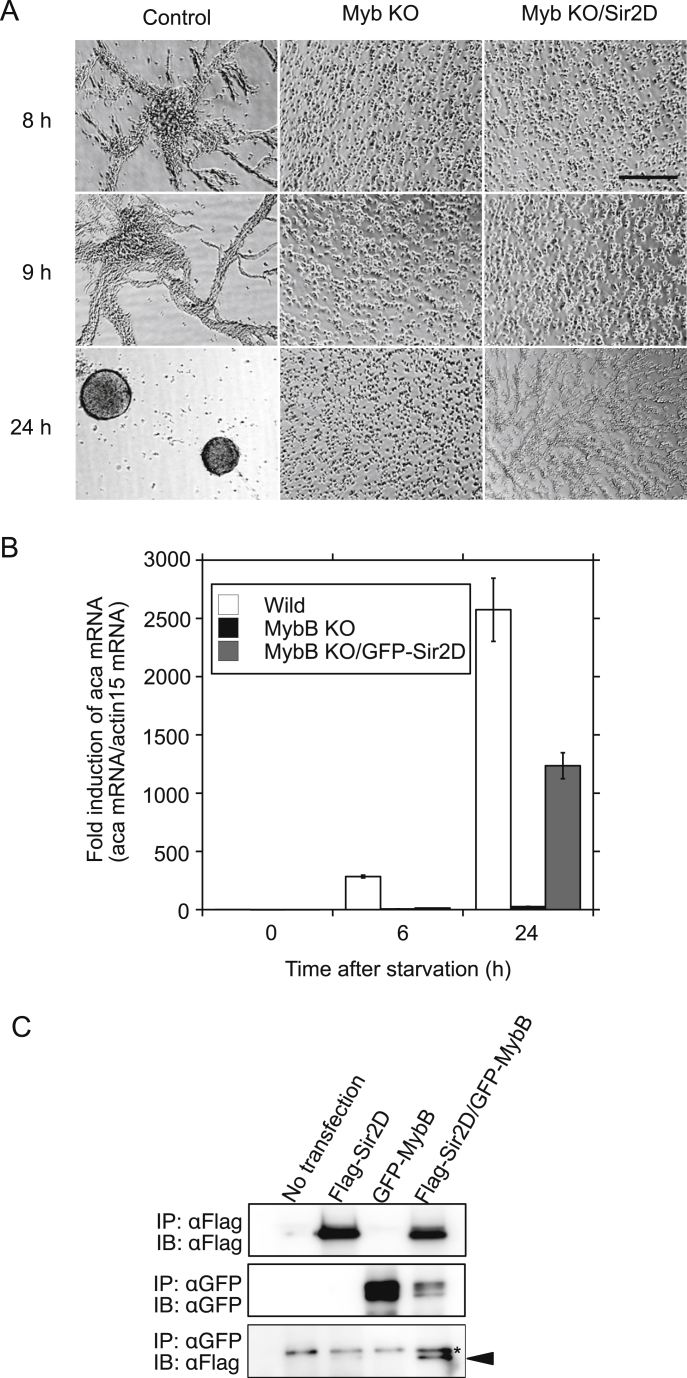
Interaction between Sir2D and MybB. (A) Cellular development of MybB KO cells. Preparation of Wild-type cells (Wild), MybB KO cells (MybB KO), and MybB KO cells coexpressed with GFP-Sir2D (MybB KO/Sir2D) were developed under submerged culture. Development was imaged 8, 9 and 24 h after starvation. Scale bar: 200 μm. (B) Alteration of mRNA expression of aca in MybB OE cells during early development upon starvation. Wild-type cells (Wild), MybB KO cells (MybB KO), and MybB KO cells coexpressed with GFP-Sir2D (MybB KO/Sir2D) were developed under submerged culture. Total RNA was extracted from the cells 0, 2, 4 and 6 h after starvation. qRT-PCR was performed using specific primers for aca or actin 15. The quantified results were normalized against actin 15 mRNA levels. Each value represents the mean ± standard error (SE) (n = 3). (C) Interaction between Sir2D and MybB. Cell lysates were prepared from wild-type Ax-2 cells (Wild) and Ax-2 cells expressing Flag-Sir2D alone, GFP-MybB alone, and Flag-Sir2D with GFP-MybB. The expression of Flag-Sir2D protein is shown as detected by anti-Flag antibody immunoprecipitated by Flag antibody (IP: αFlag, IB: αFlag). Expression of the GFP-MybB protein is shown as detected by anti-GFP antibody immunoprecipitated by GFP antibody (IP: αGFP, IB: αGFP). Cell lysates were immunoprecipitated with anti-GFP antibody, and Flag-Sir2D was detected by anti-Flag antibody in the fourth column to show the interaction between Flag-Sir2D and GFP-MybB. IP, immunoprecipitation; IB, immunoblot; αFlag, anti-Flag antibody; αGFP, anti-GFP antibody. Asterisk (*) indicates a nonspecific band. Arrow indicates the precipitated Flag-Sir2D.

MybB is a homologue of the Myb-related transcription factor in *Dictyostelium* and has been identified as one of the components regulating aca expression at the growth/differentiation transition [4]. When MybB was expressed as GFP-tagged protein at the N terminus (MybB OE cells), MybB OE cells showed accelerated development upon starvation as reported previously [8]. Cell streaming was observed at 5 h, along with a significant increased in the expression of aca compared to control cells at 2 and 4 h by 9.4- and 1.6-fold, respectively (Fig. 6A and B, GFP-MybB). However, MybB expression did not affect the development of Sir2D KD cells. Sir2D KD cells coexpressed with MybB showed delayed streaming at 9 h and a reduction in aca expression at 4 h (Fig. 6A and B, GFP-MybB/Sir2D KD) compared to controls, which was different from Sir2D KD cells coexpressed with Sir2D (Fig. 5A and B). Original images of development under submerged culture are shown in Supplementary Fig. S5. The results suggest that MybB activation requires Sir2D. Next, we isolated MybB-null mutants according to the method described previously [4]. The targeting construct contains a genomic fragment that encompasses the MybB coding region from the position +32 to +2949 (+1 at the beginning of the ATG start codon) in which a BSR cassette was inserted at the SacI site (the position +1501) (Fig. 7A). The recombinant clone was identified by the presence of PCR products using two sets of primers shown (Fig. 7A and B). The disruption of MybB was further confirmed by qRT-PCR, but mybB mRNA expression was not completely missing (Fig. 7C). When MybB-null mutant cells were starved, they displayed an aggregate-less phenotype even after 24 h (Fig. 8A) [4,8]. We have previously shown that RSV had no effects on the development of MybB-null mutant cells [8]. Sir2D expression in MybB-null mutant cells also had almost no effects, although weak streaming was seen after 24 h. The original images are shown in Supplementary Fig. S6A. Both MybB-null mutant cells and the mutant cells coexpressed with Sir2D showed quite low levels of the aca expression during early development, but moderately increased expression of aca after 24 h was detected in MybB-null mutant cells coexpressed with Sir2D (Fig. 8B). Aca was also slightly induced at 24 h in MybB KO cells alone, suggesting that mybB mRNA expression was not completely missing. We tested the interaction of Sir2D with MybB by coimmunoprecipitation assay (Fig. 8C). Flag-tagged Sir2D was coexpressed with GFP-tagged MybB. When the cell lysates were immunoprecipitated with anti-GFP antibody, Flag-Sir2D was coprecipitated with GFP-MybB (Fig. 8C, Flag-Sir2D/GFP-MybB), but was not detected in the cell lysates from wild-type cells or cells expressing Sir2D alone or MybB alone. The entire images are shown in Supplementary Fig. S6B. The results suggest that Sir2D interacts with MybB.

## 4 Discussion

We have previously reported that NAM, a Sirt1 inhibitor, treatment delayed the development and decreased the expression of aca 4 h after starvation [8]. Here, we identified a Sirtuin involved in early *Dictyostelium* development upon starvation. We showed here that ectopic expression of Sir2D accelerated the initiation of streaming timing and upregulated aca expression in early *Dictyostelium* development upon starvation. When Sir2D OE cells were treated with NAM, the timing of cell streaming was restored comparable to the development of control cells against NAM treatment. However, aggregate formation of Sir2D OE cells was severely impaired by NAM treatment after 24 h. Because Sir2D KD cells showed no impairment in aggregate formation after 24 h, NAM may target other Sirtuins during a later phase of early development.

Our result suggests that Sir2D regulates aca expression through interaction with MybB upon starvation. MybB is a homologue of the Myb-related transcriptional factor in *Dictyostelium* and has been identified as being one of the components regulating aca expression at the early development upon starvation [4]. We showed that ectopic expression of MybB accelerated the timing of streaming initiation and increased the expression of aca 2 and 4 h after starvation. In contrast, both Sir2D KD cells and the cells treated with NAM showed that the timing of streaming initiation was delayed, and the aca expression was decreased 4 h after starvation. Thus, the induction of aca at 4 h is regulated by MybB and Sir2D and necessary for appropriate timing of streaming initiation. Sir2D KD did not affect the aca expression 6 h after starvation. rasC and cAMP-dependent protein kinase, PKA, are also involved in aca expression at a later phase. PKA is a central regulator of *Dictyostelium* development that mediates changes in the expression of many genes during the developmental process [18, 19]. PKA is thought to regulate aca expression upon starvation because a mutant with a deletion of PKA-C (catalytic subunit) is unable to express aca [20]. After cAMP binds to cAMP receptor cAR1 (carA), RasGTP stimulates the activity of the lipid kinase PI3K, which converts phosphatidyl inositol phosphate PIP2 to PIP3. Various proteins bind through their PH-domains to membrane patches enriched in PIP3. One of these PH-domain proteins, CRAC activates the adenylyl cyclase ACA [21, 22].

Sirt1 interacts and regulates some transcription factors and cytoplasmic proteins including p53, FOXO, PPARγ, and PGC-1α. For example, Sirt1 binds and represses genes controlled by PPAPγ, including genes mediating fat storage, to mobilize fat in white adipocytes upon starvation [13]. FOXO1 forms a transcriptional complex at the adiponectin promoter with C/EBPα. Sirt1 enhances interaction with C/EBPα [15]. Sirt1 activates PGC-1α, resulting in enhanced mitochondrial function [14, 16]. Our results suggest that Sirtuins in *Dictyostelium* interact with regulatory proteins involved in developmental processes in a similar fashion to mammalian Sirt1 preserved through the evolution.

Sirtuins are a family of NAD-dependent enzymes that deacetylate lysine residue on various proteins. We tried to detect alteration in acetylation levels of MybB. When GFP-MybB OE cells were treated with either RSV or NAM, no alteration in acetylation levels was detected in the immunoprecipitates with anti-GFP antibody using anti-acetyl-lysine antibody (Upstate, #06-933) (Supplementary Fig. S7). MybB activity may be regulated by just binding with Sir2D, or some improvement may be necessary for detection.

## Declarations

### Author contribution statement

Hideo Taniura: Conceived and designed the experiments; Performed the experiments; Analyzed and interpreted the data; Wrote the paper.

Shuhei Soeda: Conceived and designed the experiments.

Tomoko Ohta, Maya Oki, Risako Tsuboi: Performed the experiments.

### Funding statement

This work was supported in part by MEXT-Supported Program for the Strategic Research Foundation at Private Universities.

### Competing interest statement

The authors declare no conflict of interest.

### Additional information

No additional information is available for this paper.

## Figures and Tables

**Figure:**
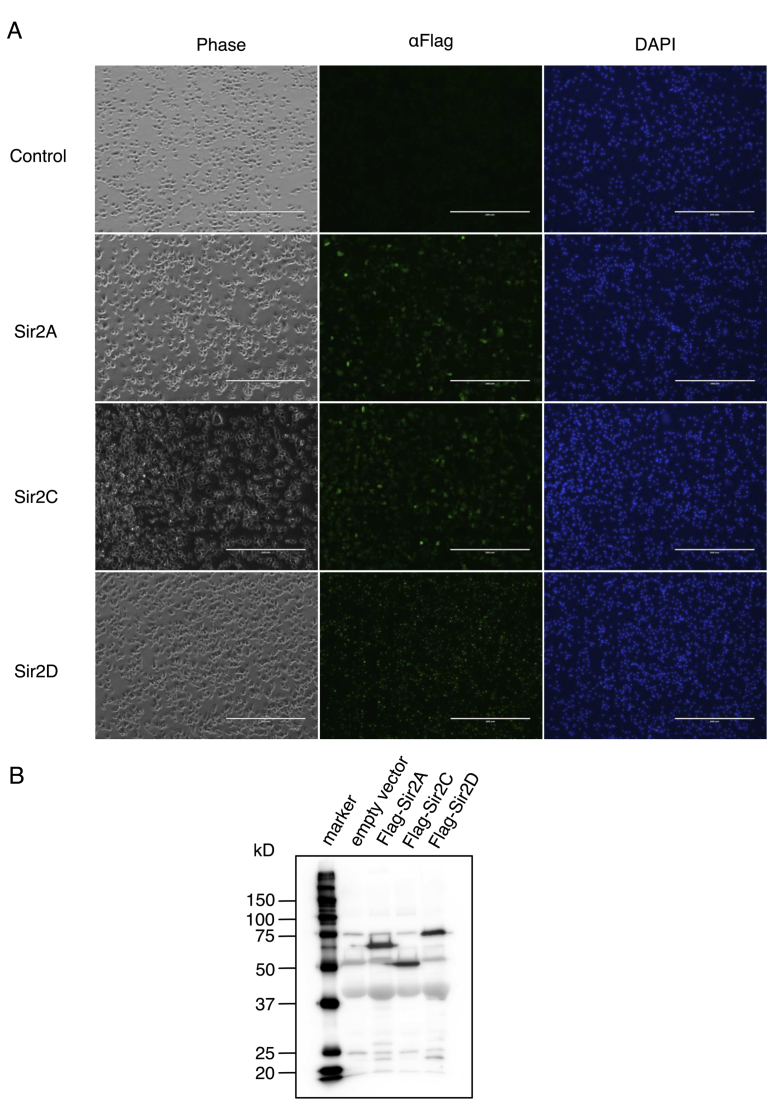
Supplementary Fig. S1.eps

**Figure:**
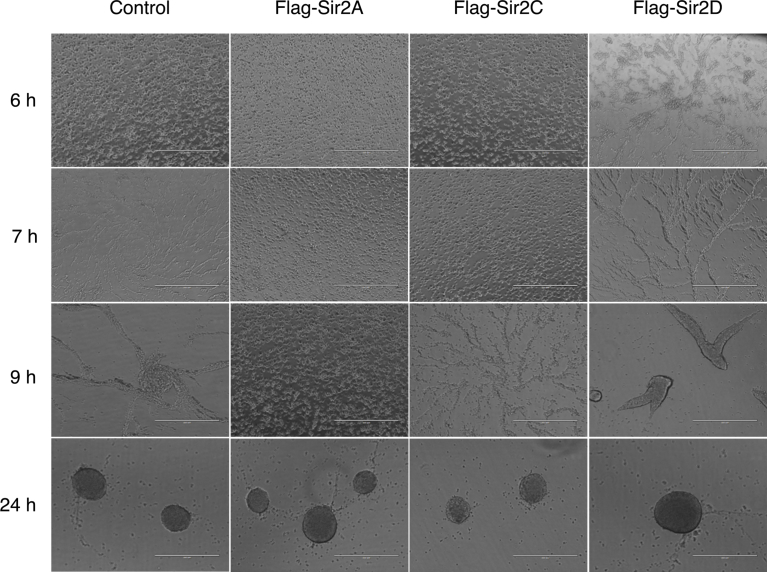
Supplementary Fig. S2.eps

**Figure:**
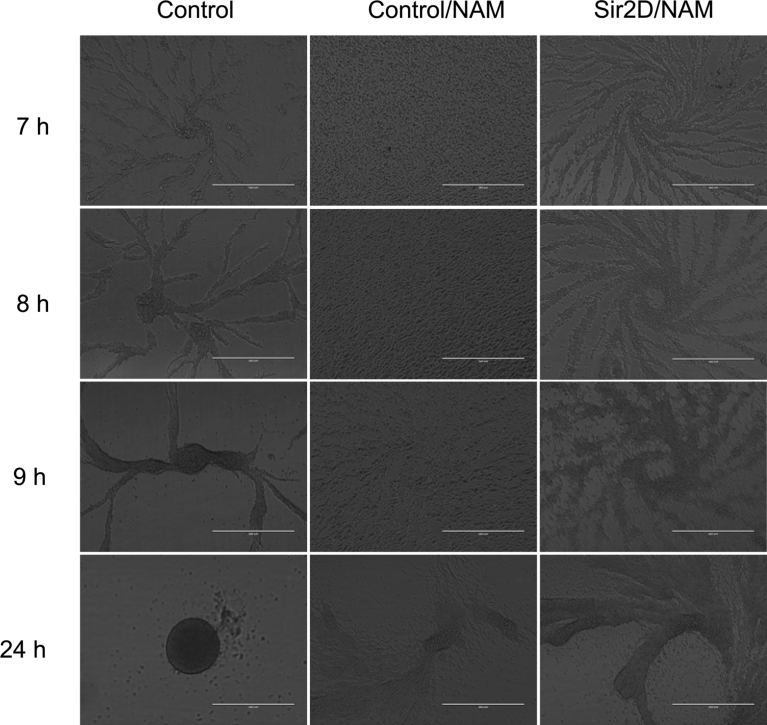
Supplementary Fig. S3.eps

**Figure:**
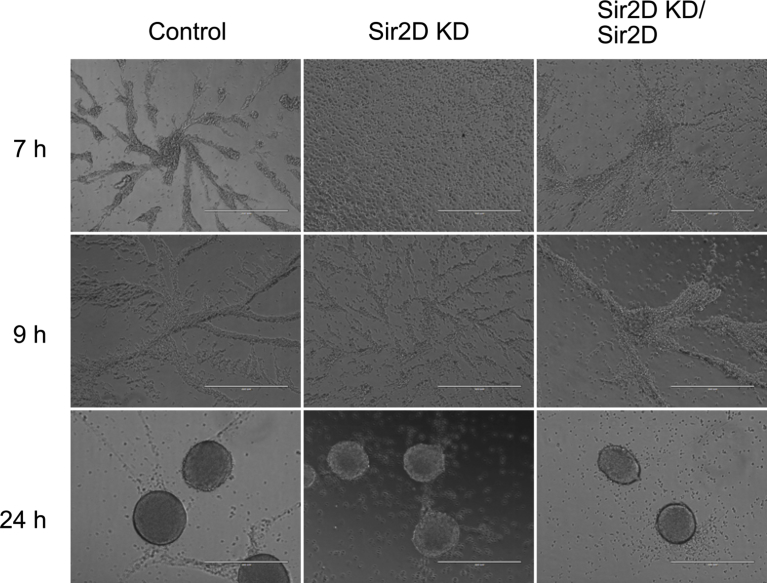
Supplementary Fig. S4.eps

**Figure:**
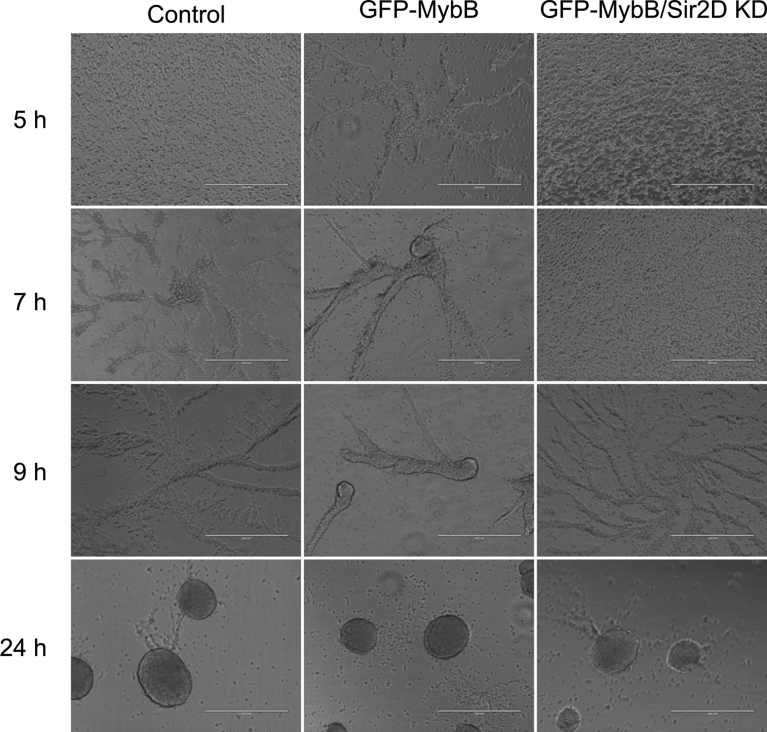
Supplementary Fig. S5.eps

**Figure:**
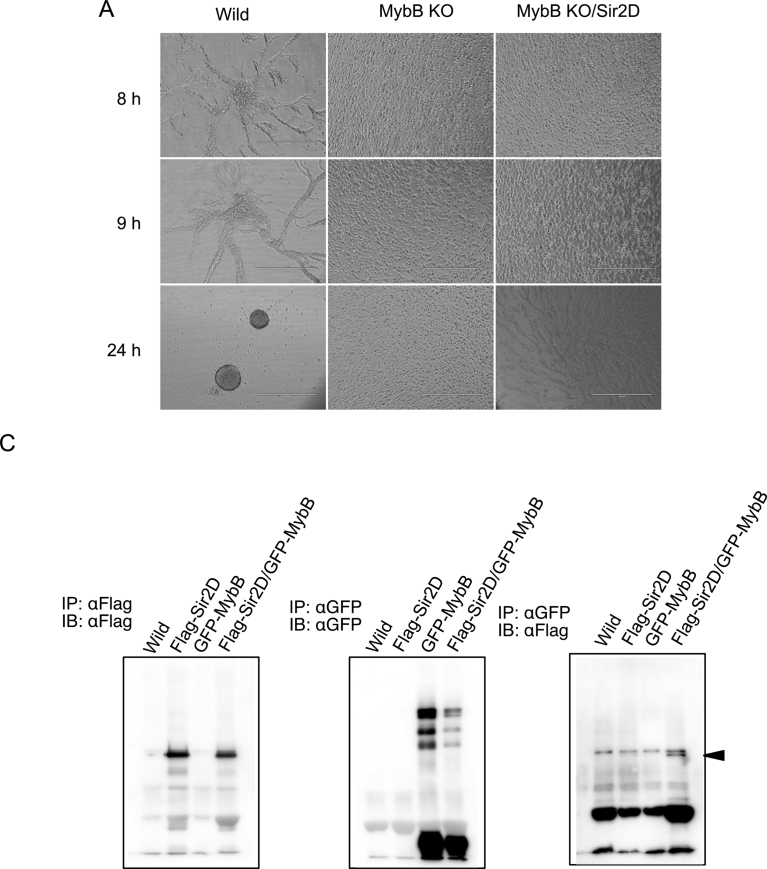
Supplementary Fig. S6.eps

**Figure:**
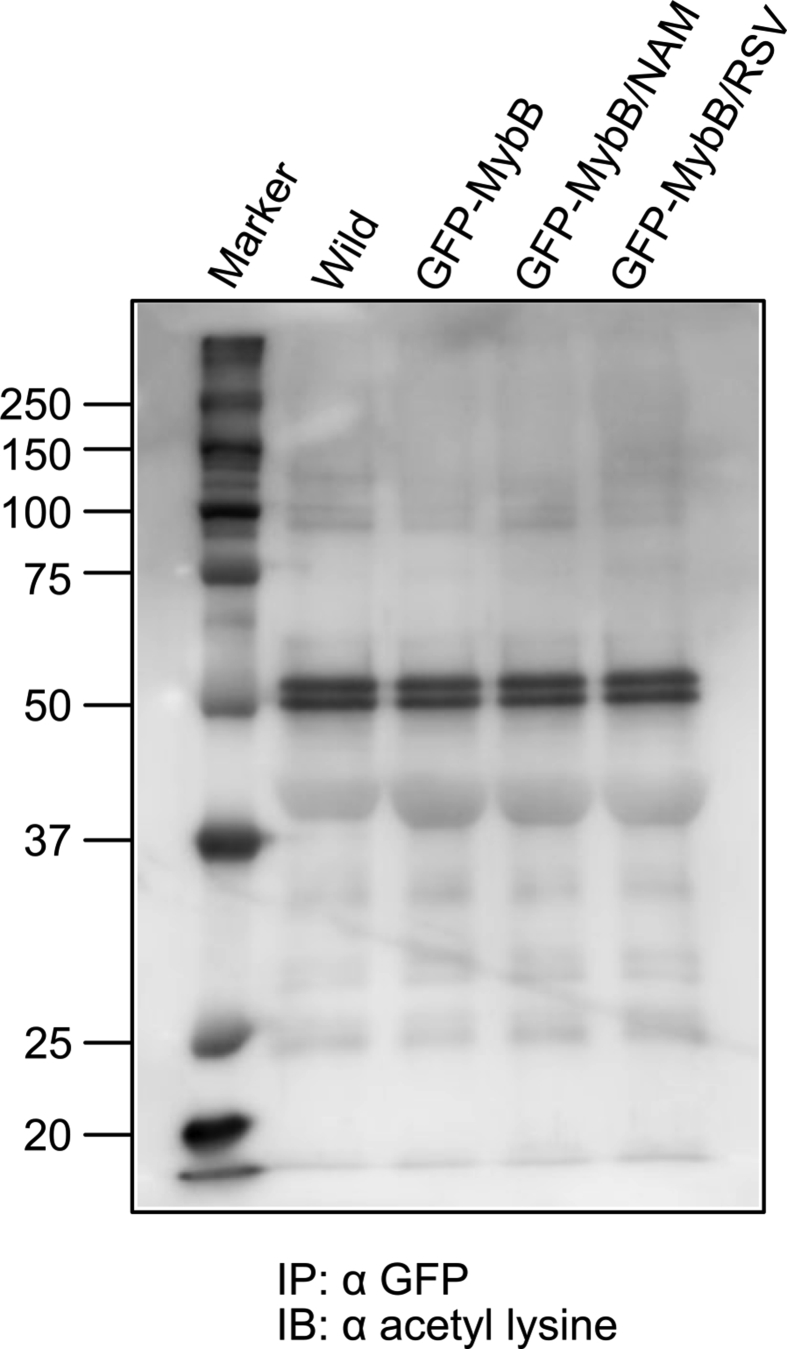
Supplementary Fig. S7.eps

## References

[1] Michan S., Sinclair D. (2007). Sirtuins in mammals: insights into their biological function. Biochem. J..

[2] Vassilopoulos A., Fritz K.S., Petersen D.R., Gius D. (2011). The human sirtuin family: evolutionary divergences and functions. Hum. Genom..

[3] Kriebel P.W., Parent C.A. (2004). Adenylyl cyclase expression and regulation during the differentiation of *Dictyostelium discoideum*. Life.

[4] Ostuka H., Van Haastert P.J.M. (1998). A novel Myb homolog initiates Dictyostelium development by induction of adenylyl cyclase expression. Genes Dev..

[5] Schaap P. (2016). Evolution of developmental signaling in Dictyostelid social amoebas. Curr. Opin. Genet. Dev..

[6] Katayama T., Yasukawa H. (2008). Analysis of Sir2E in the cellular slime mold *Dictyostelium discoideum*: cellualr localization, spatial expression and overexpression. Dev. Growth Differ..

[7] Lohia R., Jain P., Jain K., Burma P.K., Shrivastava A., Saran S. (2017). *Dictyostelium discoideum* Sir2D modulates cell-type specific gene expression and is involved in autophagy. Int. J. Dev. Biol..

[8] Soeda S., Taniura H. (2018). Sirtuin activator and inhibitor affect early Dictyostelium development upon starvation. Cell Biol..

[9] Taniura H., Tanabe N., Bando Y., Arai N. (2015). Nse1 and Nse4, subunits of the Smc5-Smc6 complex, are involved in *Dictyostelium* development upon starvation. Dev. Growth Differ..

[10] Levi S., Polyakov M., Egelhoff T.T. (2000). Green fluorescent protein and epitope tag fusion vectors for *Dictyostelium discoideum*. Plasmid.

[11] Loomis W.F. (2015). Genetic control of morphogenesis in *Dictyostelium*. Dev. Biol..

[12] Mu X., Spanos S.A., Shiloach J., Kimmel A. (2001). CRTF is a novel transcription factor that regulates multiple stages of *Dictyostelium* development. Development.

[13] Picard F., Kurtev M., Chung N., Topark-Ngarm A., Senawong T., de Oliveira R.M., Leid M., McBurney M.W., Guarente L. (2004). Sirt1 promotes fat mobilization in white adipocytes by repressing PPAR-γ. Nature.

[14] Rodgers J.T., Lerin C., Haas W., Gygi S.P., Spiegelman B.M., Puigserver P. (2005). Nutrient control of glucose homeostasis through a complex of PGC-1α and SIRT1. Nature.

[15] Brunet A., Sweeney L.B., Sturgil J.F., Chua K.F., Greer P.L., Lin Y., Tran H., Ross S.E., Mostoslavsky R., Cohen H.Y., Hu L.S., Cheng H.-L., Jedrychowski M.P., Gygi S.P., Sinclair D.A., Alt F.A., Greenberg M.E. (2004). Stress-dependent regulation of FOXO transcription factors by the SIRT1 deacetylase. Science.

[16] Nemoto S., Fergusson M.M., Finkel T. (2005). SIRT1 functionally interacts with the metabolic regulator and transcriptional coactivator PGC-1α. J. Biol. Chem..

[17] Martens H., Novotny J., Oberstrass J., Steck T.L., Postlethwait P., Nellen W. (2002). RNAi in *Dictyostelium*: the role of RNA-directed RNA polymerase and double-stranded RNase. Mol. Biol. Cell.

[18] Loomis W.F. (1998). Role of PKA in the timing of developmental events in *Dictyostelium* cells, Microbiol. Mol. Biol. Rev..

[19] Scavello M., Petlick A.R., Ramesh R., Thompson V.F., Lotfi P., Charest P.G. (2017). Protein kinase A regulates the Ras, Rap1 and TORC2 pathways in response to the chemoattractant cAMP in *Dictyostelium*. J. Cell Sci..

[20] Mann S.K.O., Brown J.M., Briscoe C., Parent C., Pitt G., Devreotes P.N., Firtel R.A. (1997). Role of cAMP-dependent protein kinase in controlling aggregation and postaggregative development in *Dictyostelium*. Develop. Biol..

[21] Insall R., Kuspa A., Lilly P.J., Shaulsky G., Levin L.R., Loomis W.F., Devreotes P. (1994). CRAC, a cytosolic protein containing a pleckstrin homology domain, is required for receptor and G protein-mediated activation of adenylyl cyclase in *Dictyostelium*. J. Cell Biol..

[22] Lim C.J., Spiegelman G.B., Weeks G. (2001). RasC is required for optimal activation of adenylyl cyclase and Akt/PKB during aggregation. EMBO J..

